# Radiotherapy with neoadjuvant chemotherapy versus concurrent chemoradiotherapy for ascending-type nasopharyngeal carcinoma: a retrospective comparison of toxicity and prognosis

**DOI:** 10.1186/s40880-017-0195-6

**Published:** 2017-03-06

**Authors:** Ji-Jin Yao, Xiao-Li Yu, Fan Zhang, Wang-Jian Zhang, Guan-Qun Zhou, Ling-Long Tang, Yan-Ping Mao, Lei Chen, Jun Ma, Ying Sun

**Affiliations:** 1 Department of Radiation Oncology, State Key Laboratory of Oncology in South China, Collaborative Innovation Center for Cancer Medicine, Sun Yat-sen University Cancer Center, 651 Dongfeng Road East, Guangzhou, 510060 Guangdong P. R. China; 2 Department of Radiation Oncology, The Fifth Affiliated Hospital of Sun Yat-sen University, Zhuhai, 519001 Guangdong P. R. China; 3 Department of Radiation Oncology, Sun Yat-sen Memorial Hospital, Sun Yat-sen University, Guangzhou, 510120 Guangdong P. R. China; 4 Department of Medical Statistics and Epidemiology & Health Information Research Center & Guangdong Key Laboratory of Medicine, School of Public Health, Sun Yat-sen University, Guangzhou, 510080 Guangdong P. R. China

**Keywords:** Nasopharyngeal carcinoma, Ascending-type, Intensity-modulated radiotherapy, Neoadjuvant chemotherapy, Concurrent chemoradiotherapy

## Abstract

**Background:**

In the era of intensity-modulated radiotherapy (IMRT), the role of neoadjuvant chemotherapy (NACT) in treating ascending-type nasopharyngeal carcinoma (NPC) is under-evaluated. This study was to compare the efficacy of NACT followed by IMRT (NACT + RT) with the efficacy of concurrent chemoradiotherapy (CCRT) on ascending-type NPC.

**Methods:**

Clinical data of 214 patients with ascending-type NPC treated with NACT + RT or CCRT between December 2009 and July 2011 were analyzed. Of the 214 patients, 98 were treated with NACT followed by IMRT, and 116 were treated with CCRT. The survival rates were assessed using Kaplan–Meier analysis, and the survival curves were compared using a log-rank test.

**Results:**

The 4-year overall survival, locoregional failure-free survival, distant failure-free survival, and failure-free survival rates were not significantly different between the two groups (all *P* > 0.05). However, patients in the CCRT group exhibited more severe acute adverse events than did patients in the NACT + RT group during radiotherapy, including leukopenia (30.2% vs. 15.3%, *P* = 0.016), neutropenia (25.9% vs. 11.2%, *P* = 0.011), and mucositis (57.8% vs. 40.8%, *P* = 0.028). After radiotherapy, patients in the CCRT group exhibited significantly higher rates of xerostomia (21.6% vs. 10.2%, *P* = 0.041) and hearing loss (17.2% vs. 6.1%, *P* = 0.023).

**Conclusions:**

The treatment outcomes of the NACT + RT and CCRT groups were similar; however, CCRT led to higher rates of acute and late toxicities. NACT + RT may therefore be a better treatment strategy for ascending-type NPC.

## Background

Nasopharyngeal carcinoma (NPC) is a common disease in South China [1]. In contrast to patients with early-stage NPC, who have 5-year overall survival (OS) rates of up to 95% [2–4], the 5-year OS rate declines to 41%–63% in patients with advanced-stage disease [5, 6]. Thus, there is a clear need to improve the treatment outcome for advanced-stage NPC.

Previous publications [7, 8] and meta-analyses [9, 10] have reported that concurrent chemoradiotherapy (CCRT) provides greater survival benefits than neoadjuvant chemotherapy (NACT) followed by radiotherapy. Additionally, a clinical trial [11] and a meta-analysis [12] found that NACT followed by CCRT was well tolerated and provided good outcomes, whereas others [13–15] question the value of concurrent chemotherapy for intensity-modulated radiotherapy (IMRT)-treated patients with locoregionally advanced NPC. These discrepancies may be partly due to the biological heterogeneity of NPC cases in the study populations. As reported by Wee et al. [16], NPC patients with predominantly advanced local disease (advanced T stage) and early-stage cervical lymph node involvement (early N stage) are classified as having the ascending type of the disease, who usually experiences local failure, whereas those with early-stage local disease (early T stage) and advanced lymph node metastases (advanced N stage) are classified as having the descending type, for whom distant failure is more common than local failure. These two types of NPC can exhibit distinct clinical-biological behaviors [17].

These previous studies did not take tumor heterogeneity into account, and differences in the numbers of cases of the ascending and descending types in the study populations could have affected the conclusions. Therefore, we included only ascending-type NPC cases in our study to avoid the effect of such tumor heterogeneity. The objective of the present study was to assess the efficacy of NACT followed by IMRT (NACT + RT) versus concurrent chemotherapy with IMRT (CCRT) on ascending-type NPC.

## Patients and methods

### Patient selection

Consecutive patients with newly diagnosed, histologically proven, non-distant metastatic, ascending-type NPC that was treated with IMRT between December 2009 and July 2011 at the Sun Yat-sen University Cancer Center (Guangzhou, China) were selected. The need for written consent was waived, whereas oral consent was obtained from the patients via telephone, as documented by telephone recording. The use of oral consent was approved by the institutional review board.

All patients completed a pretreatment evaluation, which included physical examination, chest radiography, nasopharyngeal and neck magnetic resonance imaging (MRI), abdominal sonography, and a whole-body bone scan. Patients were restaged by two radiation oncologists specializing in head and neck cancer according to the 7th edition of the Union for International Cancer Control/American Joint Committee on Cancer (UICC/AJCC) staging system, with disagreements resolved by consensus.

### Radiotherapy

The primary tumor and the upper neck area above the caudal edge of the cricoid cartilage were treated with IMRT. Target volumes were delineated according to our institutional treatment protocol [18], in agreement with the International Commission on Radiation Units and Measurements (ICRU) Reports 62 [19] and 83 [20]. The gross tumor volume (GTV) including primary nasopharyngeal tumor (GTVp) and involved lymph nodes (GTVnd) was delineated on the basis of physical examination and MRI findings. Gross disease at primary site together with enlarged retropharyngeal lymph nodes was designated GTVp; clinically-involved cervical lymph nodes, GTVnd. Two clinical target volumes (CTVs) were delineated according to the GTV: CTV1, high-risk regions encompassing GTVp plus 5–10 mm, including the entire nasopharyngeal mucosa and 5-mm submucosal region; and CTV2, low-risk regions containing CTV1 plus 5–10 mm, encompassing sites of microscopic extension and lymphatic regions. The planning target volumes (PTVs), termed PTVp, PTV1, PTV2, and PTVnd, were constructed by expanding the GTVp, CTV1, CTV2, and CTVnd, respectively, by 3 mm; a 3 mm margin was added to the brainstem and spinal cord to generate planning organ at risk volume (PRV).

The prescribed doses to PTVp, PTVnd, PTV1, and PTV2 were 66–72, 64–70, 60–63, and 54–56 Gy, respectively, in 28–33 fractions (66–70 Gy to PTVp for T1 NPC and 68–72 Gy for T2-4 NPC; 68–70 Gy to clinically-involved nodes >1 cm in diameter and 64–68 Gy to nodes ≤1 cm in diameter) [21]. The dose constraints for organs at risk (OARs) and PRVs were as described in the Radiation Therapy Oncology Group (RTOG)-0225 trial [22]. All patients were treated following a routine schedule (one fraction daily for 5 days per week).

Boost treatment was offered for selected patients at the attending physician’s discretion, usually in cases of bulky or suspected residual disease. This additional radiation was delivered by high-dose-rate intracavitary brachytherapy or external beam radiation.

### Chemotherapy

During the study period, institutional guidelines recommended neoadjuvant or adjuvant chemotherapy and/or CCRT for stage III to IVA-B NPC. For these patients, NACT was given when it was considered advantageous to reduce bulky tumors or when the waiting time for radiotherapy was considered to be longer than acceptable. The NACT consisted of cisplatin (80–100 mg/m^2^, intravenous infusion on day 1) plus 5-fluorouracil (800–1000 mg/m^2^, 120-h continuous intravenous infusion) or cisplatin (80–100 mg/m^2^, intravenous infusion on day 1) plus docetaxel (60–80 mg/m^2^, intravenous infusion on day 1) every 3 weeks for three cycles. Concurrent chemotherapy consisted of cisplatin (80–100 mg/m^2^, intravenous infusion on day 1) given in weeks 1, 4, and 7 of radiotherapy or cisplatin (30–45 mg/m^2^, intravenous infusion on day 1) given weekly during radiotherapy. Deviations from the institutional guidelines were due to organ dysfunction (suggesting intolerance to the chemotherapy) or patient refusal.

### Patient assessment and follow-up

Our primary study endpoint was OS, defined as the duration from the date of treatment initiation to the date of cancer-related death or the last follow-up. The secondary endpoints included locoregional failure-free survival (LRFFS), defined as the duration from the date of treatment initiation to the date of the first relapse in the nasopharyngeal and/or cervical region or the last follow-up; distant failure-free survival (DFFS), defined as the duration from the date of treatment initiation to the date of the first distant metastasis or the last follow-up; and failure-free survival (FFS), defined as the duration from the date of treatment initiation to the date of disease progression (local/regional recurrence or distant metastasis), death from any cause, or the last follow-up. Acute and late toxicities were documented according to the Common Terminology Criteria for Adverse Events (CTCAE) version 3.0 and/or the Radiation Morbidity Scoring Criteria of the RTOG.

The last follow-up visit was in November 2015. Patients were assessed at least every 3 months in the first 3 years and every 6 months thereafter. Routine follow-up included complete head and neck examination, nasopharyngoscopy, hematology and biochemistry profiles, chest radiography, and abdominal sonography. Follow-up neck and/or nasopharyngeal MRI was performed every 6–12 months, especially for patients with suspected tumor recurrence or radiotherapy-induced complications.

### Statistical analysis

To compare clinicopathologic features between the NACT + RT and CCRT groups, a Chi square (χ^2^) test was used for categorical variables, and a Kruskal–Wallis rank-sum test was used for continuous variables. The Kaplan–Meier method was used to estimate the rates of OS, LRFFS, DFFS, and FFS. The log-rank test was used to compare the survival rates between the NACT + RT and CCRT groups. All statistical tests were two-sided, and *P* < 0.05 was considered statistically significant. Statistical analyses were performed with R version 3.1.2.

## Results

### Patient characteristics

Between December 2009 and July 2011, 239 consecutive patients with ascending-type NPC were treated at our center. Of the 239 patients, 25 were excluded: 6 received adjuvant chemotherapy, 8 received both NACT and concurrent chemotherapy, and 11 received radiotherapy alone. Therefore, a total of 214 patients were included in the study: 98 were treated with NACT + RT, and 116 were treated with CCRT.

All patients had biopsy-proven NPC and an adequate performance status for radical treatment (Karnofsky performance score ≥70). Clinicopathologic features were well balanced between the two groups (Table 1), and there were no significant differences in the radiotherapy dose or duration. The median follow-up period was 46.8 months (range 8.9–70.3 months).

**Table 1:** Clinicopathologic features of the neoadjuvant chemotherapy plus radiotherapy (NACT + RT) group and the concurrent chemoradiotherapy (CCRT) group of patients with ascending-type nasopharyngeal carcinoma (NPC)

Variable	NACT + RT group	CCRT group	*P* value
Total	98	116	
Age (years)			0.367
Median	44	45	
Range	17–70	16–70	
Gender [cases (%)]			0.179
Male	84 (85.7)	90 (77.6)	
Female	14 (14.3)	26 (22.4)	
Histology [cases (%)]			0.795
WHO II	5 (5.1)	8 (6.9)	
WHO III	93 (94.9)	108 (93.1)	
T stage [cases (%)]^a^			0.131
T3	43 (43.9)	64 (55.2)	
T4	55 (56.1)	52 (44.8)	
N stage [cases (%)]^a^			0.118
N0	34 (34.7)	47 (40.5)	
N1	64 (65.3)	69 (59.5)	
Total radiation dose (Gy)			0.721
Median	70	69	
Range	66–72	66–72	
Overall duration of radiotherapy (days)			0.674
Median	46	47	
Range	40–49	42–51	

*WHO* World Health Organization

^a^According to the Union for International Cancer Control/American Joint Committee on Cancer staging system, 7th edition

### Treatment compliance

All patients completed the planned course of IMRT. Six patients received boost treatment after the planned course of IMRT due to the presence of gross residual disease (i.e., <100% resolution of the primary disease), as observed on follow-up MRI, or nasopharyngoscopy. Of these patients, four were treated with a brachytherapy boost (12–16 Gy at 3–4 Gy per daily fraction), and two were treated with external beam irradiation (11 Gy at 2.2 Gy per daily fraction).

In the NACT + RT group, 42 patients were treated with a PF regimen (cisplatin and 5-fluorouracil), and 56 patients were treated with a TP regimen (cisplatin and docetaxel). Six patients (6.1%) in the NACT + RT group completed only one cycle of NACT: 1 developed febrile neutropenia, 2 exhibited impaired liver function, and 3 refused further chemotherapy. The other 92 patients (93.9%) in the NACT + RT group completed 2–3 cycles of NACT. In the CCRT group, 58 patients (50.0%) were treated with cisplatin every 3 weeks during radiotherapy, and the other 58 (50.0%) were treated with weekly cisplatin during radiotherapy. Of those who were treated with cisplatin every 3 weeks, 44 (75.9%) completed two cycles, and 14 (24.1%) completed three cycles. Of those who were treated with weekly cisplatin, 52 (89.6%) completed at least four cycles. The median doses of cisplatin were 185 mg/m^2^ for the NACT + RT group and 200 mg/m^2^ for the CCRT group.

### Acute and late toxicities

In the NACT + RT group, among the severe to life-threatening (grade 3–4) hematologic adverse events that were observed during NACT, neutropenia was the most common (24 patients; 24.5%), and the most common non-hematologic adverse event was nausea/vomiting (6 patients; 6.1%). Other grade 3–4 acute adverse events included leukopenia, thrombocytopenia, hepatotoxicity, and nephrotoxicity (Table 2).

**Table 2:** Acute and late adverse events in the NACT + RT and CCRT groups

Adverse event	NACT + RT group [cases (%)]		CCRT group [cases (%)]		*P *value^†^
	Grade 1–4	Grade 3–4	Grade 1–4	Grade 3–4	
Neoadjuvant phase					
Neutropenia	61 (62.2)	24 (24.5)	NA	NA	NA
Leukopenia	54 (55.1)	20 (20.4)	NA	NA	NA
Thrombocytopenia	11 (11.2)	4 (4.1)	NA	NA	NA
Nausea/vomiting	47 (48.0)	6 (6.1)	NA	NA	NA
Hepatotoxicity	53 (54.1)	3 (3.1)	NA	NA	NA
Nephrotoxicity	37 (37.8)	1 (1.0)	NA	NA	NA
Irradiation phase					
Mucositis	98 (100)	40 (40.8)	116 (100)	67 (57.8)	0.028
Xerostomia	98 (100)	24 (24.5)	116 (100)	35 (30.2)	0.439
Skin	98 (100)	11 (11.2)	116 (100)	19 (16.4)	0.376
Nausea/vomiting	41 (41.8)	7 (7.1)	68 (58.6)	10 (8.6)	0.885
Leukopenia	43 (43.9)	15 (15.3)	71 (61.2)	35 (30.2)	0.016
Neutropenia	36 (36.7)	11 (11.2)	75 (64.7)	30 (25.9)	0.011
Thrombocytopenia	15 (15.3)	3 (3.1)	24 (20.7)	7 (6.0)	0.157
Hepatotoxicity	28 (28.6)	1 (1.0)	40 (34.5)	2 (1.7)	0.864
Nephrotoxicity	22 (22.4)	0 (0)	32 (27.6)	0 (0)	0.789
Post-irradiation phase					
Xerostomia	98 (100)	10 (10.2)	116 (100)	25 (21.6)	0.041
Hearing loss	34 (34.7)	6 (6.1)	69 (59.5)	20 (17.2)	0.023
Neuropathy	24 (24.5)	2 (2.0)	37 (31.9)	1 (0.9)	0.761
Neck tissue damage	9 (9.2)	0 (0)	15 (12.9)	0 (0)	0.867
Dysphagia	1 (1.0)	0 (0)	2 (1.7)	0 (0)	0.998

Comparison of the incidence of grade 3–4 events between the two groups

*NA* not applicable

^†^ *P* values were calculated using a Chi square test

Acute toxicities during radiotherapy were well tolerated by both groups. During radiotherapy, the rates of grade 3–4 adverse events were higher in the CCRT group than in the NACT + RT group (mucositis: 57.8% vs. 40.8%, *P* = 0.028; leukopenia: 30.2% vs. 15.3%, *P* = 0.016; and neutropenia: 25.9% vs. 11.2%, *P* = 0.011) (Table 2).

Common late adverse events included xerostomia, hearing loss, neuropathy, and neck tissue damage. The rates of grade 3–4 xerostomia and hearing loss were significantly lower in the NACT + RT group than in the CCRT group (10.2% vs. 21.6%, *P* = 0.041; 6.1% vs. 17.2%, *P* = 0.023). The rates of all other adverse events were not significantly different between the two groups (all *P* > 0.05) (Table 2).

### Treatment outcomes

**Fig. 1:**
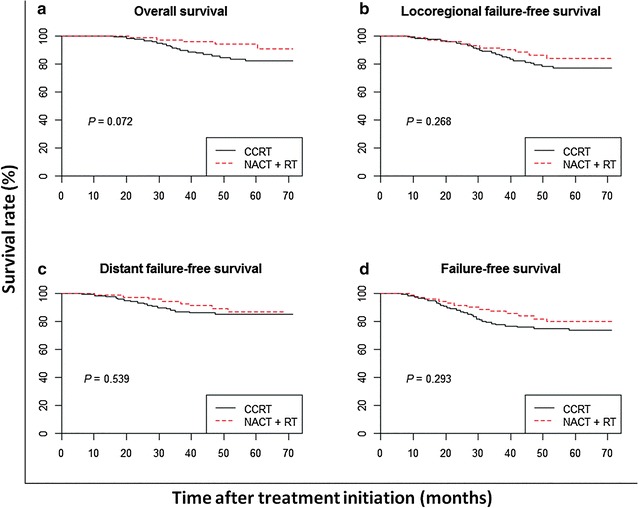
Kaplan–Meier estimates of the survival of patients with the ascending type of nasopharyngeal carcinoma (NPC) treated with neoadjuvant chemotherapy plus subsequent radiotherapy (NACT + RT) or with concurrent chemoradiotherapy (CCRT). **a** Overall survival; **b** locoregional failure-free survival; **c** distant failure-free survival; **d** failure-free survival

The 4-year OS rates were 92.3% in the NACT + RT group and 82.1% in the CCRT group (Fig. 1a). The two groups had a similar risk of death (HR = 0.41; 95% CI 0.16–1.12; *P* = 0.072). The 4-year LRFFS rates were 87.2% in the NACT + RT group and 79.5% in the CCRT group (Fig. 1b), and the HR for the treatment effect was 0.66 (95% CI 0.32–1.38; *P* = 0.268). Fourteen patients in the NACT + RT group and 25 in the CCRT group developed locoregional failure. The 4-year DFFS rates were 84.7% in the NACT + RT group and 81.4% in the CCRT group (Fig. 1c), and the HR for the treatment effect was 0.77 (95% CI 0.33–1.78; *P* = 0.539). Thirteen patients in the NACT + RT group and 23 in the CCRT group developed distant metastases. The 4-year FFS rates were 76.3% in the NACT + RT group and 73.2% in the CCRT group (Fig. 1d), and the HR for the treatment effect was 0.70 (95% CI 0.37–1.36; *P* = 0.293). Twenty-four patients in the NACT + RT group and 32 in the CCRT group developed locoregional failure and/or distant metastases.

## Discussion

In the current study, we retrospectively compared the efficacy of NACT + RT with the efficacy of CCRT on ascending-type NPC. The results showed that NACT + RT for ascending-type NPC provided a favorable outcome in terms of 4-year OS, LRFFS, DFFS, and FFS rates. In consistent with our results, Lin et al. [15] and Qiu et al. [23] investigated patients with locoregionally advanced NPC who were treated with IMRT after NACT and demonstrated that NACT + RT produced a superb outcome in terms of OS, LRFFS, DFFS, and FFS rates in these patients. Our results further suggested that CCRT offered no significant survival benefit when compared with NACT + RT.

The treatment outcome with the application of IMRT has been demonstrated in terms of locoregional control in NPC patients [24]. The 3-year LRFFS and OS rates of NPC patients were 90%–95% and 80%–85%, respectively [25–28]. Improved locoregional control was anticipated with improved dose coverage of GTV and CTV. Therefore, the effect of chemotherapy on locoregional control in NPC patients treated with IMRT remained uncertain. Our results demonstrated that for patients with ascending-type NPC, CCRT provided no significant improvement in terms of LRFFS when compared with NACT + RT; the 4-year LRFFS rate was, in fact, higher in the NACT + RT group (87.2% vs. 79.5%), though the difference was not significant (*P* = 0.268). Lai et al. [29] found that LRFFS was significantly longer for patients who underwent IMRT than for patients who underwent two-dimensional conventional radiotherapy (2D-CRT). Compared with 2D-CRT, IMRT generates more conformal dose coverage in the target volume and therefore results in better local control. Thus, it is possible that the improved local control rates obtained with IMRT eliminated the contribution of concurrent chemotherapy to LRFFS. Furthermore, significant NACT-induced shrinkage of the tumor prior to radiotherapy increases the margin of safety between the tumor and irradiation volumes, as reported by Teo et al. [30], which could reduce the locoregional recurrence rate in patients with locoregionally advanced NPC.

Although the main advantage of NACT is reduction of distant metastases [31], the NACT + RT group did not exhibit a significantly lower rate of distant metastases compared with the CCRT group in the present study. This observation may be interpreted as follows. First, the tendency in this study was to give NACT to patients when it was considered advantageous to reduce bulky tumors or when the waiting time for radiotherapy was considered to be longer than acceptable. For those patients, tumor volumes were relatively large despite the same T stage. However, previous studies have revealed that the primary tumor volume is closely related to disease progression in NPC [32, 33]. Guo et al. [34] demonstrated that a large tumor volume was predictive of a poor prognosis and was associated with distant metastasis in NPC patients treated with IMRT. Second, this analysis may have been affected by the smaller size of the NACT + RT group compared with that of the CCRT group [98 (45.8%) vs. 116 (54.2%)].

Clearly, treatment-induced adverse events could limit efficacy in patients with locoregionally advanced NPC. In this study, specific adverse events were associated with each of the two treatment protocols. Compared with the NACT + RT group, the CCRT group suffered significantly more acute adverse events such as neutropenia, leukopenia, and mucositis during radiotherapy. These findings are consistent with those reported in previous studies that used similar irradiation techniques [35, 36]. There was also a significant difference between our two groups in terms of late adverse events: patients in the CCRT group exhibited significantly more xerostomia and hearing loss than patients in the NACT + RT group. Two underlying reasons for these findings could be as follows: first, concurrent chemotherapy may increase the sensitivity of normal tissue to radiotherapy-related injury, and second, the total radiation dose to nearby organs may be reduced after the primary tumor has been shrunken by NACT.

Compared with the CCRT group in the present study, Kong et al. [37] showed lower rates of severe irradiation-related mucositis and xerostomia in advanced NPC patients treated with NACT + CCRT. In the current study, only the patients with ascending-type NPC, predominantly advanced local disease (T3-4) with early-stage cervical lymph node involvement (N0-1), were selected, whereas Kong et al. [37] did not take into account the variability of the tumor itself. With ascending-type NPC, patients have high radiation doses to OARs due to the advanced T stage and large target volume. For example, despite the same stage (stage IV) and the same treatment for patients with T1N3 and T4N0 NPC, the adverse events might be markedly different.

## Conclusions

Compared with NACT + RT, CCRT did not significantly increase the 4-year OS, LRFFS, and FFS rates of patients with ascending-type NPC, and CCRT was associated with higher rates of severe adverse events. These results suggest that NACT + RT may be a more suitable treatment strategy for ascending-type NPC. However, our study was limited by the small sample size and the use of retrospective analysis. A prospective randomized trial will be needed to confirm the findings.
